# The Neuroprotective Potential of Betalains: A Focused Review

**DOI:** 10.3390/plants14070994

**Published:** 2025-03-21

**Authors:** Cristina Ştefănescu, Oliviu Voştinaru, Cristina Mogoşan, Gianina Crişan, Georgeta Balica

**Affiliations:** 1 Department of Pharmaceutical Botany, Iuliu Hatieganu University of Medicine and Pharmacy, 23 Gh. Marinescu Street, 400337 Cluj-Napoca, Romania; cstefanescu@umfcluj.ro (C.Ş.); gcrisan@umfcluj.ro (G.C.); bgeorgeta@umfcluj.ro (G.B.); 2 Department of Pharmacology, Physiology and Physiopathology, Iuliu Hatieganu University of Medicine and Pharmacy, 6 L. Pasteur Street, 400349 Cluj-Napoca, Romania; cmogosan@umfcluj.ro

**Keywords:** betalains, betanin, indicaxanthin, neuroprotective, neurodegenerative diseases

## Abstract

Betalains are natural, hydrophilic pigments present in a variety of plants from the order Caryophyllales, extensively used as non-toxic food colorants and antioxidants. In recent decades, betalains have been intensively researched, with numerous studies confirming their anti-inflammatory, antioxidant, antimicrobial, and antinociceptive properties. More recently, due to a significant increase in the aging population worldwide, there has been growing interest in the study of preventive effects of betalains on age-related, degenerative brain diseases. The aim of this review is to evaluate the potential neuroprotective role of betalains in the prevention of neurodegenerative diseases like Alzheimer’s disease and Parkinson’s disease, as well as other types of neurodegenerative and ischemic brain injuries. Preclinical in vivo and in vitro pharmacological studies investigating the neuroprotective effects of betalains are reviewed, with a focus on the putative mechanisms of action. Available studies in humans are also presented.

## 1. Introduction

Betalains are hydrophilic pigments naturally occurring in plants, with nitrogen atoms as part of their basic structure. Due to their biosynthesis from amino acids, similar to alkaloids, these compounds are also known as chromoalkaloids [1,2]. Betalains are classified into two main structural groups: betacyanins (red-violet in color) and betaxanthins (yellow-orange in color). Their color results from the presence of resonating double bonds in their structure [3]. Therefore, these compounds are known for the intense colors they confer to the different parts of plants in which they are found but also for their antioxidant properties [4].

Betalains are very important in the taxonomy of higher plants [5]. They are important secondary metabolites found in many species belonging to the order Caryophyllales, where they replace anthocyanins, one of the most abundant pigment categories in plant species [6]. Despite their similarities to anthocyanins in terms of plant organ distribution and physiological functions, betalains have distinct structures and synthetic pathways [7].

Betalains have shown promising economical potential as non-toxic food colorants, being inexpensive and easy to obtain from multiple natural sources. The dietary intake of betalains has been associated with a positive impact on human health [6]. Thus, betalains may prove to be a promising alternative therapy for oxidative stress, inflammation, and dyslipidemia-related diseases like atherosclerosis, hypertension, and cancer [8,9], additionally demonstrating antimicrobial and antinociceptive activity [6]. Their antioxidant and anti-inflammatory properties could also be important for other, less-explored protective effects in neurodegenerative diseases in which oxidative damage, inflammatory processes, and the accumulation of misfolded proteins represent key pathogenetic mechanisms [10,11].

In recent decades, an increased life expectancy has led to a growing segment of aged individuals, frequently affected by neurodegenerative diseases like Alzheimer’s disease (AD) and Parkinson’s disease (PD), but also by other types of neurodegenerative and ischemic brain diseases [12]. Although several pharmacological treatments for neurodegenerative diseases are available, they fail to provide optimal disease control and can cause serious adverse effects [12]. Therefore, the use of natural, inexpensive, and well-tolerated betalains as nutraceuticals or drug candidates with potential neuroprotective properties could offer a promising solution for aging patients with neurodegenerative diseases. In the last decade, several review articles have focused on betalains, but most have concentrated on other biological properties of these natural compounds, only briefly mentioning their protective effects against neurodegenerative processes [6,8]. Hence, this review explores in-depth the potential neuroprotective role of betalains, presenting important pharmacokinetic and stability aspects, available preclinical and human studies, as well as mechanistic and in silico data for the main compounds, where available.

## 2. Betalains and Their Sources

### 2.1. Chemical Structure and Classification of Betalains

**Figure 1:**
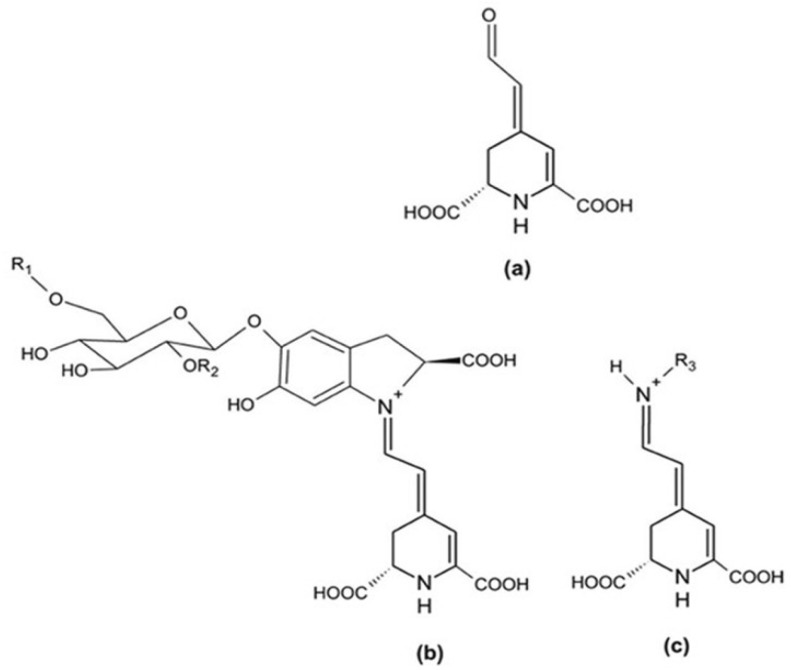
General structures of betalamic acid (**a**), betacyanins (**b**), and betaxanthins (**c**). Betanin: R1 = R2 = H; R3 = amine or amino acid group (Adapted from [16]).

Betalains are hydrophylic molecules composed of a nitrogenous core structure and betalamic acid [4-(2-oxoethylidene)-1,2,3,4-tetrahydropyridine-2,6-dicarboxylic acid] (Figure 1) [1,13,14].

The betalain class contains a wide array of compounds. Based on the nature of the betalamic acid moiety and light-absorption properties, they are generally classified into two structural groups: betacyanins (red-violet pigments) and betaxanthins (yellow-to-orange pigments) [1,14,15,16,17,18]. Up to date, the structures of about 80 plant betalains have been well characterized [18,19].

According to several authors [2,20,21], there are four groups of betacyanins: betanin-type, amaranthin-type, gomphrenin-type, 2-descarboxybetanin-type, and two betaxanthin groups: amino acid-derived conjugate group and amine-derived conjugate group (Table 1).

### 2.2. Occurrence in Nature and Role of Betalains in Plants

Betalains are a category of taxonomically relevant compounds, being known to be produced in three divergent lineages of organisms: the flowering plant lineage—only by species belonging to the order Caryophyllales; the fungal lineage—several genera in the phylum Basidiomycota (*Amanita*, *Hygrocybe*, and *Hygrophorus*); and the bacterial species—*Gluconacetobacter diazotrophicus*, a proteobacterium first described in roots and stems of sugarcane with no evident relationship with plants of the order Caryophyllales but found to be able to synthesize betalains in cultures supplemented with L-DOPA [6,22,23]. Furthermore, the biolistic introduction of DNA constructs for transient overexpression of two different dihydroxyphenylalanine (DOPA) dioxygenases (DODs) and feeding of DOD substrate (L-DOPA) induced betalain (both betaxanthins and betacyanins) production in cell cultures of *Solanum tuberosum* L. and petals of *Antirrhinum majus* L. [24].

For many decades, the order Caryophyllales just included the taxa (11 families) characterized by a free central placentation (Centrospermae), perisperm, and curved embryos (Cronquist and Thorne classification); all the species within Centrospermae were known to synthesize betalains, with the only exception of those belonging to Caryophyllaceae and Molluginaceae families [1,25]. Molecular systematic studies have added numerous further families to the order Caryophyllales, among them Polygonaceae, Plumbaginaceae, Droseraceae, Nepenthaceae, etc. [1]. As a result, the Angiosperm Phylogeny Group (APG 1998) presented Caryophyllales with an expanded taxon concept in which the so-called “core Caryophyllales” included the traditionally recognized Centrospermae and their segregated families [25]. The actual APG classification, namely APG IV, indicates 38 families within the order Caryophyllales, 28 of them included in “core Caryophyllales” [26]. Most families within the “core Caryophyllales” are betalain-pigmented, with the exception of Caryophyllaceae, Molluginaceae sensu stricto, Kewaceae, Limeaceae, Macarthuriaceae, and Simmondsiaceae, which have been reported to produce anthocyanins. Betalains have never been detected or reported in any of these six anthocyanic lineages, and anthocyanins have never been detected within betalain-producing species; however, other flavonoid-derived compounds, such as proanthocyanidins, can be found in the seed coat of some betalain-pigmented species (e.g., Spinacia) [22].

Betalains are produced in chloroplasts and are essential for their integrity [2]. After being synthesized, they accumulate in the cell vacuoles, dissolved as bis-anions, mainly in epidermal and subepidermal tissues [8,27]. Betalains are found in vegetative and re-productive elements: edible parts of plants as fruits or tubers but also in the bark, bracts, seeds, leaves, flowers, and stems [14].

The function of betalains in plants is diverse. Like other plant pigments prevalent in flowers and fruits, they likely play an important role in attraction, either of pollinators for plant propagation through pollen transfer or frugivores for fertilization or dispersion of indigestible seeds [9,28].

Betalains are also likely to participate in plant defense against various biotic and abiotic stress cues. As important plant-specialized metabolites, they have strong antioxidant properties and can scavenge oxygen free radicals; therefore, their activity in defense against abiotic stress is considered to be mediated through their strong antioxidant capacity [7,18].

Betalains are involved in plant photoprotection as “chemical shields” for the prevention of photooxidative damage, their production being upregulated when plants are exposed to light or UV radiation [7,8,18]. Increased betalain accumulation under drought and salt stress conditions coupled with induced expression of betalain-related genes indicate a role in protection against these stress factors. This coincides with their occurrence in the Caryophyllales, an order with dominance in arid and semi-arid regions, and habitation of saline and alkaline soils through families Aizoaceae (ice-plant family), Portulacaceae (purslane family), and Cactaceae (cacti family) [18]. Betalains function as a repelling signal to deter herbivores, for increasing pathogen resistance, and improving viral defense, for providing higher tolerance to heat stress, or as osmoregulators by modeling the amino acid pool from betaxanthins [8].

### 2.3. Sources of Betalains

Multiple edible sources of betalains are available, including red and yellow beetroot (*Beta vulgaris* L. ssp. *vulgaris*), colored Swiss chard (*Beta vulgaris* L. ssp. *vulgaris* convar. *cicla* var. *flavescens*), leafy or grainy amaranth (*Amaranthus* sp.), cactus fruit (*Opuntia* sp. and *Hylocereus* sp.), and quinoa grains (*Chenopodium quinoa* Willd.) [6,8,29].

The red beetroot (*B. vulgaris*) is the major commercially exploited crop to produce betalains, cultivated especially in North and Central America and in Great Britain. The betacyanin yield identified in the root was 30–60 mg/100 g fresh weight (betanin and isobetanin being present), while the betaxanthin yield was 16.3 mg/100 g fresh weight (vulgaxanthin I and miraxanthin V being identified) [6,29,30].

The prickly pear (*Opuntia* spp.) is native to Mexico, being spread and cultivated across the world in Latin America, South Africa, and the Mediterranean [29,31]. *Opuntia ficus-indica* (L.) Mill. is one of the most cultivated species for fruit production [31]. The yield of betacyanins and betaxanthins reported in the prickly pear fruit pulp was 13.4 mg/100 g fresh weight and 24.3 mg/100 g fresh weight, respectively [30].

The red pitahaya or red dragon fruit (*Hylocereus lemairei* (Hook.) Britton and Rose, synonym *H. polyrhizus* (F.A.C. Weber) Britton and Rose) is widely cultivated in Malaysia, China, Okinawa, Israel, and Vietnam, and is also mentioned as a popular Aztec fruit, being native to southern Mexico and Central America [10,29,32]. The fruit peels were found to contain 73 mg/100 g fresh weight betacyanins (with betanin and isobetanin being detected) and no betaxanthins [30].

Amaranth is a rediscovered “new” crop. It was an ancient crop, cultivated as a staple food of the ancient Aztecs. It is cultivated as a minor crop in regions of Central and South America, Asia, and Africa. China, where it is used for food purposes, occupies an important place in grain amaranth production in the world [5]. Betacyanins were identified in the leaves of different *Amaranthus* species, with a yield of 237 mg/100 g fresh weight in the leaves of *A. dubius*, in which amaranthine was detected [30].

Betalains are also present in red-violet varieties of quinoa (*Chenopodium quinoa*), which was the traditional grain crop of the prehispanic civilizations in America, being used as a feedstock, with an important economic and cultural background; recently it has gained a renewed relevance as an alternative crop to cereals due to its excellent nutritional value [33]. The amount of betalains identified in quinoa grains is 30.23 mg/100 g for betacyanins (amaranthine and betanin being indicated), and 9.58 mg/100 g for betaxanthins (dopaxanthin and dopamine-betaxanthin being present) [30].

An estimated 681.388 tons of betalains represents the minimum value of betalains that could be produced in the world from beetroot, prickly pear, pitaya, and amaranth [30], but new potential sources of betalains could emerge using genetic engineering and biotechnologies. Thus, betanin was obtained into engineered rice endosperm as a health-promoting food additive to increase health benefits of rice as well as provide a potential source of raw material for commercial supplement production [4]. The cloning of the enzyme 4,5-DOPA-dioxygenase from *Gluconacetobacter diazotrophicus* in an expression vector and the subsequent heterologous expression in *Escherichia coli* cultures have led to the start-up of a biotechnological production system of individual betalain pigments [34]. A *Saccharomyces cerevisiae* strain capable of producing betanin from central metabolism was created, using an optimized and engineered version of *Beta vulgaris* CYP76AD1, and 4,5-DOPA-dioxygenase enzyme from *Mirabilis jalapa* L. optimized for *S. cerevisiae* [35].

## 3. Chemical Stability and Bioavailability of Betalains

Betalain stability is affected by numerous pigment specific and external factors. They are relatively sensitive to environmental factors such as heat, pH, and oxygen [13,36]. Temperature is the most crucial factor influencing betalain stability both during food processing and storage [17]. Betalains are sensitive to heat and degrade at temperatures above 50 °C [18], with a considerable degradation between 50 and 75 °C [37].

**Figure 2:**
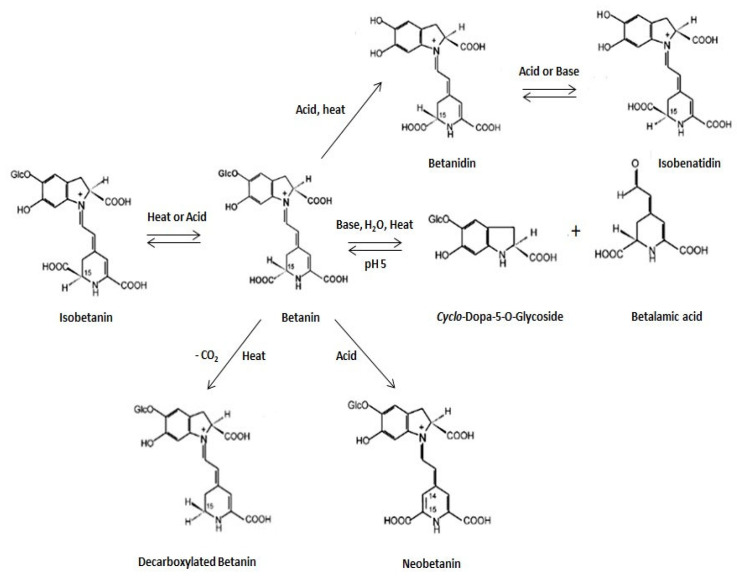
Betanin degradation pathways and resulting products (Adapted from [8]).

During heat processing, betanin may be degraded by cleavage (by heat or acids), decarboxylation, or isomerization [8] (Figure 2).

Betalains are stable within a pH range of 3 to 7 [8,13,16], being more stable than anthocyanins which have an optimum pH range of 5 to 6 [21]. Strongly acidic or alkaline conditions cause structural changes in the molecules of betalains [17,38,39]. Betalain pigments are considered to be unstable in the presence of oxygen. The stability is decreased linearly when oxygen concentration is increased. In addition to oxygen, hydrogen peroxide is also a cause of pigment degradation. However, the betalain stability is considered to be increased in the presence of nitrogen atmosphere [17,36]. Illumination was also reported to deteriorate betalain stability [16,17]. The combined effects of oxygen and light have shown a 28.6% degradation of betanin after storing pigment solutions under air or nitrogen, with or without exposure to light, for a six-day period at 15 °C [40].

The main challenge on betalain administration by food or pharmaceutical products is their instability against environmental factors, requiring the use of stabilization and controlled release methods. The addition of additives such as antioxidants, chelators, pectin, guar gum, or β-cyclodextrin can improve betalain stability. Encapsulation, which is the coating of betalains with relatively inert materials, increases their useful life, masks undesirable flavors and odors, and offers a controlled release [30,41]. Betalain encapsulation methods are represented by the introduction or synthesis in cyclodextrins; spray-drying and freeze-drying using mainly maltodextrin or mixtures of maltodextrin with other polymers such as gelatin, soy protein, mucilage from cladodes of nopal cactus (*O. ficus-indica* (L.) Mill.), pectin, Arabic gum, sodium alginate, adsorption on macroporous resins, double emulsions, ionic gelation, hydrogels, nanoliposomes obtained using soy oil, soy protein, alginate, or lecithin [30,42,43].

The bioavailability of betalains was investigated by a limited number of studies using in vitro and in vivo models both in humans and laboratory animals. Initial in vitro studies using perfused isolated organ techniques suggested that betalains could be absorbed in the intestine and metabolized in the intestinal wall and also the liver [44]. Moreover, a study on Caco-2 cells, which simulated a functional intestinal barrier, proved the capacity of betanin and indicaxanthin to pass through intestinal epithelial cells [45]. However, the passage of betalains through the digestive tract can cause losses in the amount of the absorbed active substances due to physiological factors like interactions with gut microbiota or enzymatic degradations. An in vitro study from 2020 which used a simulated gastrointestinal digestion showed that only 27% of betaxanthins were present at the end of the digestion process [46].

In humans, the administration of a single dose of 300 mL beetroot juice containing 120 mg betalains to volunteers caused the urinary excretion of small amounts of betanin and isobetanin (0.5–0.9% of the administered dose), thus proving the oral absorption of the active substances [47]. In another study, the administration of 16 mg betanin to human volunteers produced detectable plasmatic concentrations of the active compound with a maximum plasma concentration (Cmax) of 0.2 nmol/mL after 3.1 h, thus showing a relatively low bioavailability. The terminal elimination half-life (T_1/2_) was 0.94 h for betanin, suggesting a high clearance rate [48]. A recent pharmacokinetic study in humans from 2024, showed that betalains are intensively metabolized during their digestive transit by decarboxylation and dehydrogenation reactions leading to a primarily fecal excretion of metabolites, the renal route contributing in a smaller part to the total excretion of betalains [49].

The influence of long-term administration of betalains on pharmacokinetic parameters was studied on 24 healthy volunteers who consumed 0.7 mg/kg betalains for 6 weeks, every morning after breakfast. Afterwards, betalains were analyzed in plasma and urine by HPLC/MS techniques. Twelve betalain molecules and also their metabolites were found in plasma and urine of the volunteers with peak levels detected in the first and second week of administration. The study proved that a long-term administration of betalains causes a stabilization of their concentration in the physiological fluids of humans [50].

The ability of betalains to cross the blood–brain barrier (BBB) is a key parameter for the development of a neuroprotective effect; however, it was rarely investigated. A pharmacokinetic study from 2015 showed that the oral administration of indicaxanthin in rats in doses compatible with a dietary consumption in humans generated variable brain concentration of the compound, proving its ability to cross the blood–brain barrier. Thus, the oral administration of 2 µmol/kg indicaxanthin in rats generated measurable concentrations in the rat brain after 1 h, with a peak of 20 ± 4 ng indicaxantin/whole brain, 2.5 h after the administration of the compound. Afterwards, indicaxanthin disappeared from the rat brain within 4 h, following a first-order kinetics [51]. A subsequent study investigated more thoroughly the brain distribution of indicaxanthin after oral administration in rats. A single oral administration of 2 µmol/kg indicaxanthin to Wistar rats, followed by HPLC analysis of various brain tissues, showed an accumulation of the active compound in cortex, hippocampus, diencephalon, brainstem, and cerebellum, one hour after the administration, further proving the ability of betalains to cross the blood–brain barrier [52].

The relatively low bioavailability of betalains could be improved by recent formulations using nanotechnology. Thus, betanin administered in the form of liposomes showed an increased chemical stability and bioavailability in a rodent model of diabetes [53], another study from 2023 confirming that betanin nanoparticles administered to rats in a model of brain ischemia–reperfusion were able to cross blood–brain barrier and to generate sufficient concentrations of the active compound inside brain tissue [54].

## 4. Neuroprotective Potential of Betalains

Our review searched the published literature between 2010 and 2024 using PubMed, Scopus, and Web of Knowledge databases. Only full-text articles in English were included in this work. After the removal of duplicates, articles written in other languages, and studies which used red beetroot juice instead of individual betalains, we identified 12 preclinical studies investigating the protective effects of betalains against neurodegenerative diseases and other types of neuronal injuries.

### 4.1. Preclinical Studies

The preclinical studies designed for the evaluation of neuroprotective effects of betalains used in vivo and in vitro experimental models for Alzheimer’s disease, Parkinson’s disease, other neurodegenerative processes, and also ischemia–reperfusion-induced neuronal injuries (Table 2).

**Table 2:** Preclinical studies (in vivo and in vitro) investigating potential neuroprotective effects of betalains.

No.	Experimental Model	Substances and Doses/Route of Administration (In Vivo)	Main Findings	Reference
	**Models of Alzheimer’s disease**			
1.	Transgenic *Caenorhabditis elegans*	50 µM betanin and isobetanin	Reduction in beta-amyloid-induced toxicity; reduction in amyloid-β42 aggregation	[55]
2.	In vitro assessment of AchE	12.5–400 µM betanin and glycine betaine	An 86.6% and 92.9% inhibition of acetylcholinesterase with IC_50_ of 1.271 and 1.203 µM	[56]
3.	AlCl_3_-induced Alzheimer in SD rats	10–20 mg/kg betalain/oral	Reduction in cognitive deficit; reduction in inflammatory cytokines in the brain	[57]
4.	Scopolamine-induced Alzheimer symptoms in Wistar rats	25–50 mg/kg betanin/oral	Reduction in learning deficit in passive avoidance tests	[58]
5.	Trimethyltin-induced neurodegeneration in mice	50–100 mg/kg betanin/oral	Prevention of hippocampal CA1 neural degeneration; increase in choline acetyltransferase activity; improvement of spatial learning and memory deficits	[59]
	**Models of Parkinson’s disease**			
6.	Rotenone-induced Parkinson in mice	50–100 mg/kg betanins/s.c	Improvement of motor tests; reduction in substantia nigra degeneration in treated animals	[60]
7.	Rotenone-induced Parkinson in mice	100–200 mg/kg betanin/oral	Prevention of substantia nigra, striatum and motor cortex neural degeneration	[61]
8.	In vitro assessment of 6-hydroxydopamine toxicity in PC12 cells	1–200 µM betanin	Reduction in PC12 cells apoptosis; reduction in the progression of neural death	[62]
	**Other models of neurodegeneration and brain injuries**			
9.	High fat-diet fed mice	0.86 mg/kg indicaxanthin/oral	Reduction in neuronal apoptosis by decreasing the expression of neuroinflammatory proteins and ROS and nitrogen species	[63]
10	Ischemia–reperfusion induced neural injuries in ICR mice	50–100 mg/kg betanin/oral	Reduction in brain infarctions and hippocampal white matter lesions	[64]
11.	Paracetamol and diclofenac induced neurotoxicity in rats	25 mg/kg betanin/oral	Protection against drug-induced neurotoxicity; amelioration of biochemical and histopathological brain changes	[65]
12.	D-galactose-induced neurotoxicity in mice	50–100 mg/kg betanin/oral	Reversal of D-galactose-induced learning and memory impairments	[66]

In Alzheimer’s disease, beta-amyloid protein can aggregate in the brain, disrupting normal synaptic communication and leading to premature neural death. Therefore, the ability of betalains to specifically reduce the aggregation of beta-amyloid was investigated in a transgenic model, using the nematode *Caenorhabditis elegans* modified to express the human amyloid gene (*CL2006*) and subsequently to accumulate amyloid-β42 fragments in its muscles, leading to paralysis [67]. The administration of betanin and isobetanin to the transgenic nematodes in concentrations of 2, 10, and 50 µM, delayed and reduced the paralysis of *Caenorhabditis elegans*, by the inhibition of beta-amyloid-β42 fragment aggregation which was proved by transmission electron microscopy and nuclear magnetic resonance analysis [55]. Other experimental models used toxins like AlCl_3_ or trimethyltin in order to induce neurodegenerative processes. AlCl_3_ can accumulate mainly in the hippocampus, favoring the development of beta-amyloid aggregates and neurofibrillary tangles by increasing the oxidative stress, while trimethyltin can create a region-specific neurodegeneration, selectively affecting hippocampal neurons important for memory [68]. In AlCl_3_ model, the oral administration of 10–20 mg betanin to Sprague Dawley rats for four weeks caused an improvement in learning and memory capacity evaluated in radial arm maze and Morris water maze tests [57]. In trimethyltin-induced neurodegeneration model, the oral administration of 50–100 mg/kg betanin to ICR mice for two weeks, caused an improvement of spatial learning and memory deficits in behavioral tests and protected hippocampal CA1 neurons against degeneration by antioxidant mechanisms [59]. Also, in Alzheimer’s disease, the degeneration of cholinergic neurons has been correlated with the loss of memory [69]; therefore, a possible capacity of betalains to increase synaptic concentrations of acetylcholine was investigated. An anticholinesterase effect of betalains was confirmed by in vitro techniques, using Ellman’s spectrophotometric assay. The method used acetylcholine chloride as substrate for acetyl-cholinesterase enzyme and decreasing concentrations of betanin (400–12.5 µM), the absorbance being recorded at 405 nm. Dose response analysis showed a strong enzyme inhibition (86.6% for betanin and 92.9% for glycine betaine), with IC_50_ values of 1.271 μM and 1.203 μM for betanin and glycine betaine [56].

In a different experimental model of Alzheimer’s disease, the administration of 25–50 mg/kg betanin for 9 consecutive days to Wistar rats with scopolamine-induced memory deficit caused significant improvements. The treated animals exhibited a reduction in memory and learning deficits in novel object recognition and passive avoidance tests. The study proved by histopathological techniques that betanin reduced the mitochondrial dysfunction and tissue injuries in hippocampal area, acting by potent antioxidant mechanisms [58].

For Parkinson’s disease, an experimental model used rotenone, a potent inhibitor of complex I of the mitochondrial electron transport chain in order to cause a loss of dopaminergic neurons in the substantia nigra, and subsequently a motor dysfunction [70]. The oral administration of betanin 100 and 200 mg/kg for 6 weeks in ICR mice proved significant neuroprotective effects evaluated by motor tests like Rotarod and by immunohistochemical methods. Betanin reduced neural degeneration in the substantia nigra and striatal regions of the brain and increased the density of tyrosine-hydroxylase in the striatum, causing also an increase in reduced glutathione, catalase, and superoxide dismutase activities in the brain [61]. An in vitro experimental model used PC12 cells treated with the toxic compound 6-OHDA (6-hydroxydopamine), which can induce apoptosis of dopaminergic neurons in rodents but also in humans. By flow cytometry techniques, it was shown that a pretreatment of PC12 cells with betanin 1–200 µM for 24 h inhibited cell apoptosis induced by 6-OHDA (100 µM). Additionally, betanin reduced reactive oxygen species (ROS) production in the treated cells, proving to be a possible solution for the prevention of neural degeneration in Parkinson’s disease [62].

Other models of neurodegeneration used a different approach. A study from 2024 showed that the administration of a high fat diet in rodents increased neural damage by augmenting the amount of reactive oxygen and nitrogen species. A daily oral administration of 0.86 mg/kg indicaxanthin for 4 weeks in mice proved significant neuroprotective effects due to the reduction in expression of neuro-inflammatory proteins and genes (TNF-α, IL-6, COX-2) [63].

In a model of drug-induced neurodegeneration caused by toxic doses of paracetamol and diclofenac in rats, the oral administration of 25 mg/kg betanin improved biochemical and histological modifications in brain tissues [65]. An experimental model of accelerated aging used D-galactose to cause memory and learning impairments due to increased oxidative stress. The oral administration of 50–100 mg/kg betanin in senescent mice reversed the memory deficit determined in behavioral tests (water maze), acting by an inhibition of lipid peroxidation [66].

Betalains also proved a significant capacity to attenuate ischemia–reperfusion injuries, which are involved in the pathogenesis of stroke, a major cause of death and major complications worldwide. In a preclinical study, the oral pre-treatment with 50–100 mg/kg betanin of male ICR mice subjected to a surgically induced cerebral ischemia and reperfusion proved strong protective effects. The treated animals showed a significant reduction in brain infarctions and CA1 and hippocampal white matter degeneration, acting by an inhibition of lipid peroxidation and boosting of GSH and CAT activity [64].

Although multiple types of preclinical studies were employed in the evaluation of neuroprotective potential of betalains including in vitro, in vivo, and transgenic models, the translation of the results to human medicine is often difficult. The vast majority of the described in vivo models used rodents as experimental animals and various substances as disease inducers, being able to reproduce initial biochemical events in the development of neurodegenerative processes but not all the steps observed in humans. The only transgenic model used a nematode species, very distant to humans in evolutionary terms. Therefore, the introduction of other transgenic models using rodents and macaques in future studies focusing on neurodegenerative diseases could better reproduce human pathogenetic processes. Additionally, the use of new biochemical markers (serum tau proteins), recent functional neuroimaging techniques like positron emission tomography (PET), or computational artificial intelligence-assisted methods, could enhance the quality and translatability of preclinical data in future studies [71].

### 4.2. Studies in Humans

Our research identified only six small scale studies investigating potential neuroprotective effects of red beetroot in humans, useful in the prevention of age-related and neurodegenerative disorders, which have been published to date [72,73,74,75,76,77]. Although the studies generally showed improvements in neuroplasticity and an attenuation of cognitive decline in the enrolled participants which were mainly old individuals, the study protocols did not use individual betalains as treatment, using instead raw beetroot or beetroot juice. Hence, the favorable effects observed in the studies cannot be attributed to betalains alone, considering the complex chemical composition of red beetroot. Another major limitation of the mentioned studies was the reduced statistical power given by the limited number of patients enrolled in the trials.

Therefore, larger clinical trials with a superior statistical significance and better design which must include the administration of individual betalains are needed in order to confirm a possible neuroprotective effect of betalains and subsequently their use in the prevention of age-related neurodegenerative diseases.

### 4.3. Molecular Mechanisms of Betalains Responsible for Neuroprotective Effects

#### 4.3.1. Inhibition of β-Amyloid Aggregation

The inhibition of β-amyloid aggregation plays an important role in the neuroprotective effect of betalains against Alzheimer’s disease, where abnormal metabolism of the Aβ precursor protein (APP) by gamma-secretases leads to the formation of β-amyloid aggregates inside neurons partially responsible for the cognitive dysfunctions [12]. The molecular mechanisms responsible for the inhibition of β-amyloid aggregation are not very well described. A study from 2019 showed that betanin was able to reduce by an activation of Nrf2 pathway, the transcription of beta-site amyloid precursor protein cleaving enzyme 1 (BACE1), the enzyme responsible for the cleavage of APP to APP β, further cleaved by gamma-secretases to beta-amyloid fragments [78].

The ability of betanin and other natural compounds to inhibit beta-amyloid aggregation was also evaluated in a molecular docking study. It was found that betanin was the most active molecule, reducing the aggregation of amyloid Aβ40 fragments by altering the secondary structure of the oligomers. The active substance increased the beta-sheet content in key regions of the oligomers, rendering them less capable of aggregation [79]. A new molecular docking study from 2024 revealed that betalains interacted with the N-terminal domain of Aβ (1-42) peptide by hydrogen bonding and hydrophobic interactions, reducing the aggregation capacity of the peptide [80].

#### 4.3.2. Cholinergic Mechanisms

In Alzheimer’s disease, the cholinergic pathways are impaired causing significant memory and learning deficits. The enzyme acetylcholinesterase (AchE) breaks down synaptic acetylcholine, an important brain neurotransmitter involved in learning and cognition processes. Thus, an augmentation of cholinergic transmission by blocking the degradation of acetylcholine with acetylcholinesterase inhibitors has led to the authorization of several drugs like donepezil or rivastigmine, commonly used in the symptomatic treatment of Alzheimer’s disease [80]. Several studies investigated whether betalains were able to increase the cholinergic transmission by inhibiting acetylcholinesterase (AchE), the enzyme responsible for acetylcholine breakdown. The inhibitory activity of betalains on AchE was demonstrated in vitro, the main active compound betanin being able to cause a significant effect, comparable to donepezil (LogIC_50_ 1.287 µM vs. 1.154 µM) [56]. A recent study from 2024, using PC12 cells, also showed that pretreatment with betanin 10–50 µM, caused a significant acetylcholinesterase inhibition [81].

Cholinergic mechanism of betalains was confirmed by a molecular docking study which evaluated the interactions of 16 active substances from *Beta vulgaris* with acetylcholinesterase enzyme. The study found that betanin had a significant binding affinity towards AchE, with multiple hydrophobic interactions with several residues from the enzyme (Gly120, GlyA448, GlyA121, SerA2023, His A447). The binding energy for betanin was −22 kcal/mol, compared with the value of donepezil, the reference drug, which was −17 kcal/mol [56].

#### 4.3.3. Anti-Inflammatory Mechanisms

**Figure 3:**
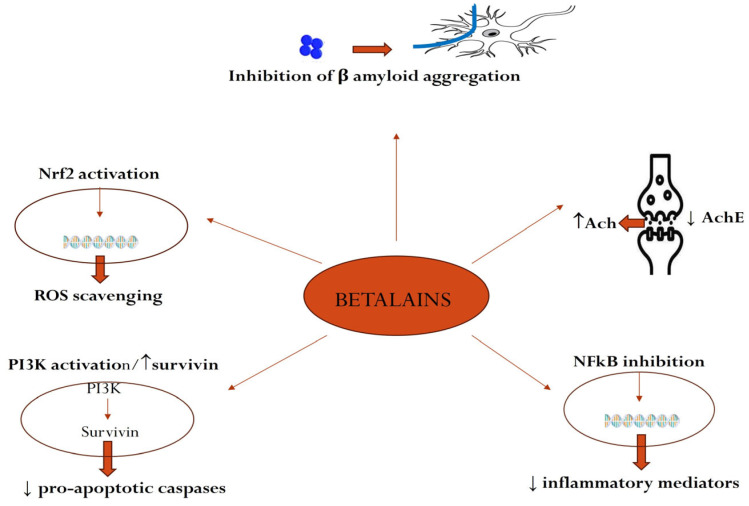
Main neuroprotective mechanisms of betalains (Ach-acetylcholine; AchE-acetylcholinesterase; NfkB-nuclear factor kB; PI3K-phosphatidylinositol 3 kinase; ROS-reactive oxygen species; Nrf2-nuclear factor erythroid 2-related factor).

The anti-inflammatory effect of betalains is complex, being partially responsible for the favorable effects in neurodegenerative disorders. At nuclear level, the transcription factor NF-kB plays a center role in inflammation, promoting the activation of multiple pro-inflammatory cytokines. Normally, NF-kB is associated with inhibitory protein IkBα, but in inflammation, they dissociate, and NF-kB is able to enter the cell nucleus to exert its role [82]. An inhibitory effect of betanin on the nuclear factor NF-kB with the reduction in the expression of pro-inflammatory cytokines like IL-1, IL-6 and TNF-alpha and a subsequent inhibition of neuroinflammation was demonstrated [57]. Another study showed that betanin reduced the gene expression of NF-kB in the brain and promoted the phosphorylation of the inhibitory protein IkBα, thus reducing the activation of the nuclear factor [64] (Figure 3).

Moreover, the capacity of natural compounds like flavonoids and isocoumarins to directly inhibit cyclooxygenase (COX) and lipoxygenase (LOX) enzymes in order to reduce the synthesis of important pro-inflammatory mediators (prostaglandins and leukotrienes) derived from arachidonic acid pathways, was proved by previous studies [83,84,85]. Betalains are also capable to inhibit the synthesis of inflammatory mediators, acting by similar mechanisms. Thus, a direct inhibition of COX-2 and LOX by betanin was demonstrated in vitro by a study which proved a higher affinity of betanin for COX-2 isoenzyme with a percentage of inhibition of 97% [86,87]. In addition, betanin and indicaxanthin have the capacity to inhibit intercellular adhesion molecules like ICAM-1, expressed on endothelial cells but also on immune cells, with important roles in inflammation due to its effect of facilitating leucocyte adhesion to vascular endothelium [88].

The anti-inflammatory mechanisms of betalains were also studied using in silico models. A molecular docking study investigated the ability of betalains to bind to COX and LOX enzymes, finding that the compounds interacted with Tyr-385 and Ser-530 residues close to the active site of COX [47]. Another recent molecular modeling study using induced-fit docking (IFD) and binding pose meta dynamics (BPMD) techniques investigated the ability of indicaxanthin to inhibit NF-kB. It was found that indicaxanthin was able to bind and inhibit the active form of human large cytoplasmic protein complex (IKK) with a subsequent sequestration of NF-kB and reduction in its’ pro-inflammatory effects [89]

#### 4.3.4. Reduction in Oxidative Stress

Excessively produced reactive oxygen species and an insufficient response from endogenous antioxidant systems may lead to an increased oxidative damage to DNA molecules, proteins, or lipids from cell membranes, being directly involved in the pathogenesis of various conditions including neurodegenerative diseases [90]. Beetroot is an exceptionally rich source of antioxidants, betalain pigments, especially betacyanins being amongst the most active compounds capable of reducing oxidative stress [91,92]. The administration of betalains proved significant protective effects against oxidative stress in several in vitro or in vivo experimental models. Several in vitro studies confirmed the radical scavenging properties of betalains against ABTS+, lipoperoxyl or DPPH^−^. The results showed that betanin was 1.5–2 times more efficient than anthocyans as a radical scavenger [93]. Other research was focused on nuclear and gene modulatory mechanisms of the antioxidant effect of betalains, finding that the activation of Nrf2-ARE pathway may also be responsible for the antioxidant effect. Nuclear factor erythroid 2 (Nrf2) is a transcription factor with key roles in antioxidant defense which is normally kept in an inactive state by the association with the inhibitory protein Keap1. The dissociation from Keap1 activates Nrf2 which enters the cell nucleus, binds to a specific DNA element called antioxidant responsive element (ARE), and activates several genes codifying antioxidant enzymes [94]. A study on cell cultures showed that betanin was able to facilitate the dissociation of Nrf2 from Keap1, then its translocation from cytoplasm to cell nucleus, with downstream activation of several genes encoding antioxidant enzymes [95]. Another study proved that betanin was able to inhibit the gene expression of NOX-4, an important generator of reactive oxygen species but also to regulate the expression of glutathione S-transferase A, reducing the oxidative stress in specific organs [96].

The identified nuclear mechanism was confirmed by a molecular docking study which found that betanin can interact with Keap1 and also can interact with the Silent information regulator 2-related protein 1 (SIRT1), causing a subsequent reduction in the degradation of Nrf2 [97].

#### 4.3.5. Reduction in Apoptosis

Apoptosis is a key feature of a variety of neurodegenerative disorders, leading to the loss of specific neurons. The ability of betanin to influence the molecular mechanisms of apoptosis was investigated on rat pheochromocytoma cells (PC12 cells). It was found that betanin was able to reduce the phosphorylation of stress-activated protein kinase/c-Jun N terminal kinase 46/54 (SAPK/JNK 46/54) and increase the phosphorylation of phosphatidyl-inositol 3 kinase (PI3K). PI3K activation leads to the inhibition of pro-apoptotic proteins and promotes anti-apoptotic factors like survivin. Subsequently, an increase in the intracellular amount of survivin inhibited the activation of several caspases with strong anti-apoptotic effects [62]. The above-mentioned mechanism by which betalains can cause the reduction in apoptosis was further confirmed by a recent study from 2024, which found that pretreatment of PC12 cells with betanin in concentrations of 10–50 µM caused the reduction in cell death, decreasing the level of poly (ADP-ribose) polymerase (PARP) and cytochrome c by caspase inhibition [81].

## 5. Safety Profile of Betalains

Betalains are important natural compounds used in food and pharmaceutical industry. A lead betalain representative, betanin was approved as a red food colorant (EEC No. E162) by the European Union [98]. Several toxicological tests were performed to investigate the safety profile of betalains.

The acute oral toxicity of betalains was evaluated in Wistar rats. The administration of an extract containing 0.23% betacyanins (indicaxanthin, phyllocactin, betanin, and isobetanin) to rats by oral route for 15 days in doses of 0.5 to 5 g/kg did not cause any change in body weight or any gross morphological change [99]. Another short-term toxicity study in rats showed that the administration of 2.000 mg/kg betalains in the diet for 7 days did not cause any changes in body weight, food behavior, or any gross pathological alteration [100]. In a subchronic toxicity test, the oral administration of beetroot juice corresponding to 53 mg/kg/day betacyanins and 26 mg/kg/day betaxanthins to Wistar rats for 28 days did not cause any change in the level of liver superoxide dismutase, glutathion peroxidase, catalase, or plasma protein carbonyl percentage compared to control animals [101].

Betalains did not show genotoxicity or mutagenic capacity in studies using the bacteria *Salmonella typhimurium*. Thus, a betalain concentration of 500 to 2500 micrograms/plate did not cause any mutagenic effect in the Ames test using five strains of *S. typhimurium* [99]. Genotoxicity was also evaluated in rat bone marrow. The administration of 0.2 and 50 mg/kg/day betanin to Wistar rats for 7 days did not change the frequency of micronuclei apparition in bone marrow cells [102].

In humans, the administration of red beetroot juice in doses up to 500 mL in a single dose in a clinical setting did not cause any significant adverse effect [75]. Occasionally, the administration of beet pigments can cause beeturia (excretion of colored urine) but the phenomenon is not considered pathological [44]. The allergenicity of betalains was also evaluated, highlighting that because of the lack of reports of allergic reactions and the widespread use of beetroot red (betanin) it can be concluded that betanin is not a safety concern regarding allergies and hypersensitivity reactions [98,103].

The available studies suggest that betalains are safe for human consumption, although embryotoxicity and teratogenicity studies are not available.

Natural compounds like betalains can influence liver enzymatic systems similar to those involved in drug metabolism with the posible development of drug interactions. A recent study using in vitro techniques investigated the effects of betanin on six important liver enzymes belonging to CYP450 system, involved in the metabolism of a multitude of drugs. It was found that betanin showed no effects on CYP1A2, CYP2B6, CYP2C9, and CYP2C19, exhibited only a weak inhibitory effect on CYP2D6, but showed a significant inhibitory effect on CYP3A4 with IC_50_ of 20.97 μM [104]. Therefore, caution is advised when associating betanin with drugs strongly metabolized by CYP3A4 (benzodiazepines, antidepressants, anticonvulsants, calcium channel blockers, antidiabetics, anticoagulants) due to a potential increase in their plasmatic concentrations [105]. In case of drugs commonly used in the treatment of neurodegenerative diseases, no specific reports of interactions with betalains have been found so far.

## 6. Conclusions

Currently, the available drugs against neurodegenerative diseases are not able to adequately control disease progression, often being limited by the development of adverse reactions. Therefore, significant research efforts have been made to identify new molecules of natural origin, with good bioavailability and tolerability, that may be effective in the prevention of neurodegenerative disorders and other neuronal injuries. Among a variety of tested natural compounds, betalains have shown a favorable potential to become successful nutraceuticals and drug candidates due to their good safety profile and the availability of multiple natural sources in the plant kingdom.

Betalains are considered multi-targeted natural compounds, capable of modulating a variety of pathogenetic processes, demonstrating significant neuroprotective potential. In Alzheimer’s disease, betalains can reduce amyloid aggregation, increase cholinergic transmission, and reduce neuro-inflammation. In Parkinson’s disease, betalains act through strong antioxidative, antiapoptotic, and anti-inflammatory mechanisms. Additionally, betalains can reduce the extent of other types of neuronal injuries like drug-induced or ischemia–reperfusion brain lesions. Although existing preclinical studies show promising results, other experimental models using transgenic animals or modern brain imaging techniques are required for better translation of these results into human medicine. Moreover, additional clinical trials using individual betalains and larger cohorts of patients are needed to fully ascertain the importance of the neuroprotective potential of betalains.

## Figures and Tables

**Table 1:** Classification of the main betalains in plants [2,8,13,14,15,16,17].

Betacyanins
*Betanin group*: Betanin (betanidin 5-O-glucoside); Phyllocactin (6′-O-malonyl-betanin); 2′-Apiosyl-phyllocactin; 2′-[5″-O-(E)-Feruloylapiosyl]-betanin; Hylocerenin (6′-O-(3″-hydroxy-3″-methyl)-betanin)
*Amaranthin group*: Amaranthin (betanidin 5-O-glucuronosylglucoside); Iresinin I (hydroxymethylglutaryl-amaranthin); Celosianin I, II (coumaroyl and feruloyl-amaranthin)
*Gomphrenin group*: Gomphrenin I (betanidin 6-O-glucoside); Gomphrenin II (coumaroyl derivative of gomphrenin I); Gomphrenin III (feruloyl derivative of gomphrenin I); Betanidin 6-O-(6-O-hydroxycinnamoyl)-sophoroside derivatives
*2-Descarboxy-betanin group*: 2-Descarboxy-betanidin; 2-Descarboxy-betanin; 6′-O-malonyl-2-descarboxy-betanin
**Betaxanthins**
*Amino acid-derived conjugate group*: Portulacaxanthin II (L-tyrosine-betaxanthin); Portulacaxanthin III (glycine-betaxanthin); Tyrosine-betaxanthin; Tryptophan-betaxanthin; Dopaxanthin
*Amine-derived conjugate group*: 3-Methoxytyramine-betaxanthin; γ-aminobutyric acid-betaxanthin; Indicaxanthin;Vulgaxanthin I

## Data Availability

Not applicable.

## Abbreviations

6-OHDA	6-hydroxydopamine
AchE	acetylcholinesterase
APP	amyloid beta precursor protein
ARE	antioxidant responsive element
BACE1	beta-site amyloid precursor protein cleaving enzyme 1
COX2	cyclooxygenase 2
FGF	fibroblast growth factor
FRAP	ferric-reducing antioxidant power
IC50	half-maximal inhibitory concentration
ICAM1	intercellular adhesion molecule 1
IL1	interleukin 1
IL6	interleukin 6
KEAP1	Kelch-like ECH-associated protein 1
LOX	lipooxygenase
NF-kB	nuclear factor-kB
Nrf2	nuclear factor erythroid 2-related factor
PI3K	phosphatidylinositol 3 kinase
PARP	poly (ADP-ribose) polymerase
SAPK	stress-activated protein kinase
SIRT1	silent information regulator 2-related protein 1
VCAM	vascular cell adhesion molecule-1
TNFα	tumor necrosis factor alpha
